# Supplementary material for: Investigating pedigree- and SNP-associated components of heritability in a wild population of Soay sheep
Source: Heredity (Edinb). 2024 Feb 10;132(4):202–10. doi: 10.1038/s41437-024-00673-6 (PMC10997785; doi:10.1038/s41437-024-00673-6)
Supplement: Supplementary file 1 — Supplementary Tables 1 - 7 [file 41437_2024_673_MOESM1_ESM.pdf]

	Causal gene	Chromsome	Gene start bp	Gene end bp	MAF
Male horn type	RXFP2	10	29,454,677	29,502,617	0.471*
Female horn type	RXFP2	10	29,454,677	29,502,617	0.471*
Coat colour	TYRP1	2	80,602,298	80,623,437	0.49
Coat pattern	ASIP	13	63,237,431	63,242,627	0.226**

Supplementary Table 1: Causal genes, the location of the causal genes (chromsome, start bp and end bp) and minor allele frequencies (MAF) of the monogenic traits.

* Equilibrium frequency

** The self-type coat pattern has been linked to up to five haplotypes which contain any of three recessive variants in the *ASIP* gene. The MAF reported here is the frequency of all five recessive haplotypes combined.

Model	Threshold	Parameter	Neonate<br>Birth weight
Model 1		h^2_{GRM}	0.0504848 (0.0189423)
Model 2	t=0.05	h^2_{pop}	0.0310285 (0.0246469)
		h^2_{kin}	0.0343825 (0.0282527)
		h^2_{pk}	0.0654110
	t=0.1	h^2_{pop}	0.0453195 (0.0230756)
		h^2_{kin}	0.0100933 (0.0261491)
		h^2_{pk}	0.0554128
Model 4		h^2_{ped}	0.0782698 (0.0274258)

Model	Threshold	Parameter	Lamb<br>August weight	Foreleg length	Hindleg length	Metacarpal length	Jaw length
Model 1		h^2_{GRM}	0.0836002 (0.0227376)	0.1401640 (0.0250416)	0.1406110 (0.0265688)	0.3198500 (0.0378536)	0.2543860 (0.0357230)
Model 2	t=0.05	h^2_{pop}	0.0685738 (0.0310563)	0.1102290 (0.0316158)	0.1165390 (0.0351703)	0.2954930 (0.0471020)	0.2155440 (0.0426954)
		h^2_{kin}	0.0263117 (0.0362078)	0.0450873 (0.0326143)	0.0402105 (0.0385569)	0.0420406 (0.0509113)	0.0844032 (0.0484278)
		h^2_{pk}	0.0948855	0.1553163	0.1567495	0.3375336	0.2999472
	t=0.1	h^2_{pop}	0.0835192 (0.0279068)	0.1182790 (0.0288547)	0.1261280 (0.0318137)	0.3004040 (0.0428648)	0.2292180 (0.0394293)
		h^2_{kin}	0.0001715 (0.0318213)	0.0399467 (0.0292312)	0.0297991 (0.0345896)	0.0465531 (0.0482062)	0.0830081 (0.0470872)
		h^2_{pk}	0.0836907	0.1582257	0.1559271	0.3469571 (0.0657066)	0.3122261
Model 4		h^2_{ped}	0.0920266 (0.0307689)	0.1857650 (0.0351451)	0.1970670 (0.0396617)	0.4068190	0.3167840 (0.0619316)

Model	Threshold	Parameter	Adult<br>August weight	Foreleg length	Hindleg length	Metacarpal length	Jaw length
Model 1		h^2_{GRM}	0.2407890 (0.0341295)	0.2789680 (0.0383702)	0.4408300 (0.0408771)	0.6507510 (0.0478387)	0.5597250 (0.0511207)
Model 2	t=0.05	h^2_{pop}	0.2007170 (0.0542305)	0.2154630 (0.0520131)	0.4582100 (0.0730839)	0.6552330 (0.0877115)	0.4511790 (0.0866933)
		h^2_{kin}	0.0478639 (0.0521969)	0.0735910 (0.0468383)	0.0000009 (0.0700422)	0.0000010 (0.0887920)	0.1347760 (0.0842002)
		h^2_{pk}	0.2485809	0.2890540	0.4582109	0.6552340	0.5859550
	t=0.1	h^2_{pop}	**0.1669180** (0.0478874)	**0.1939620** (0.0454238)	0.3804200 (0.0641476)	0.6272610 (0.0753312)	0.4270150 (0.0793274)
		h^2_{kin}	0.0982557 (0.0467192)	0.1121880 (0.0407194)	0.0752228 (0.0599039)	0.0303275 (0.0720732)	0.1689700 (0.0735212)
		h^2_{pk}	0.2651737	0.3061500	0.4556428	0.6575885	0.5959850
Model 4		h^2_{ped}	0.2377390 (0.0382416)	0.3131680 (0.0431046)	0.4447200 (0.0472902)	0.6589970 (0.0582174)	0.5821470 (0.0606762)

(green shading) Indicates that the LRT gave a p value < 0.05 for the inclusion of the thresholded GRM

BOLD Indicates that the h^2_{pop} estimate does not fall within the standard error of the h^2_{GRM} estimate

Supplementary Table 2: h^2_{GRM} (h^2 with no thresholded GRM fitted), h^2_{pop} (the additive genetic variance explained by the full GRM (equivalent to h^2_{GRM} from model 1)), h^2_{kin} (the additive genetic variance explained by the thresholded GRM), h^2_{pk} (the sum of h^2_{pop} and h^2_{kin}) and h^2_{ped} (h^2 estimated using the pedigree) estimates for each polygenic trait for models 1, 2 and 4 with their respective standard errors.
Green highlight indicates that the LRT gave a p value < 0.05 for the inclusion of the thresholded GRM, whilst bolded h^2_{pop} estimates indicate that the h^2_{pop} estimate does not fall within the standard error of the respective h^2_{GRM} estimate.

			Neonate	Lamb				
			Birth weight	August weight	Foreleg length	Hindleg length	Metacarpal length	Jaw length
Model 1		h^2_{GRM}	0.0504848 (0.0189423)	0.0836002 (0.0227376)	0.1401640 (0.0250416)	0.1406110 (0.0265688)	0.3198500 (0.0378536)	0.2543860 (0.0357230)
Model 3	t=0.1	h^2_{pop}	0.0887579 (0.0355111)	0.1131290 (0.0421449)	0.1838270 (0.0447654)	0.2002460 (0.0491091)	0.3570040 (0.0644263)	0.2922570 (0.0582801)
		h^2_{kin}	0.0000009 (0.0319769)	0.0000009 (0.0385133)	0.0000009 (0.0388257)	0.0000009 (0.0445024)	0.0000009 (0.0555167)	0.0000009 (0.0502495)
		h^2_{pk}	0.0887588	0.1131299	0.1838279	0.2002469	0.3570049	0.2922579
	t=0.05	h^2_{pop}	0.0649126 (0.0256225)	0.0919911 (0.0307153)	0.1600630 (0.0333593)	0.1588910 (0.0354883)	0.3155620 (0.0486348)	0.2714230 (0.0453895)
		h^2_{kin}	0.0000009 (0.0197049)	0.0000009 (0.0246078)	0.0000009 (0.0241801)	0.0000009 (0.0271960)	0.0035804 (0.0350481)	0.0000009 (0.0338638)
		h^2_{pk}	0.0649135	0.0919920	0.1600639	0.1588919	0.3191424	0.2714239
	t=0.01	h^2_{pop}	0.0476112 (0.0194963)	0.0759578 (0.0232446)	0.1375290 (0.0257127)	0.1416860 (0.0276798)	0.3207590 (0.0397298)	0.2562250 (0.0370333)
		h^2_{kin}	0.0039096 (0.0091319)	0.0179776 (0.0141600)	0.0043647 (0.0105374)	0.0000010 (0.0120848)	0.0000010 (0.0166571)	0.0000010 (0.0157298)
		h^2_{pk}	0.0515208	0.0939354	0.1418937	0.1416870	0.3207600	0.2562260
	t=0.005	h^2_{pop}	0.0447857 (0.0188276)	0.0794582 (0.0229079)	0.1371820 (0.0252384)	0.1411110 (0.0271742)	0.3197130 (0.0387698)	0.2546130 (0.0363830)
		h^2_{kin}	0.0091967 (0.0082832)	0.0092595 (0.0105304)	0.0060356 (0.0088084)	0.0000010 (0.0091289)	0.0000010 (0.0120349)	0.0000010 (0.0113618)
		h^2_{pk}	0.0539824	0.0887177	0.1432176	0.1411120	0.3197140	0.2546140
	t=0.001	h^2_{pop}	0.0505879 (0.0189987)	0.0858074 (0.0231565)	0.1403890 (0.0251294)	0.1433450 (0.0269713)	0.3191210 (0.0380116)	0.2531430 (0.0357344)
		h^2_{kin}	0.0000010 (0.0035382)	0.0000009 (0.0060963)	0.0000010 (0.0038095)	0.0000010 (0.0063960)	0.0000010 (0.0067004)	0.0000010 (0.0060550)
		h^2_{pk}	0.0505889	0.0858083	0.1403900	0.1433460	0.3191220	0.2531440
Model 4		h^2_{ped}	0.0782698 (0.0274258)	0.0920266 (0.0307689)	0.1857650 (0.0351451)	0.1970670 (0.0396617)	0.4068190 (0.0657066)	0.3167840 (0.0619316)

			Adult				
			August weight	Foreleg length	Hindleg length	Metacarpal length	Jaw length
Model 1		h^2_{GRM}	0.2407890 (0.0341295)	0.2789680 (0.0383702)	0.4408300 (0.0408771)	0.6507510 (0.0478387)	0.5597250 (0.0511207)
Model 3	t=0.1	h^2_{pop}	0.2653940 (0.0684382)	0.2993080 (0.0652254)	0.4451120 (0.0903141)	0.6851390 (0.1121900)	0.5783910 (0.1056780)
		h^2_{kin}	0.0000009 (0.0612993)	0.0000009 (0.0537638)	0.0000010 (0.0823708)	0.0000009 (0.1071730)	0.0000009 (0.0948522)
		h^2_{pk}	0.2653949	0.2993089	0.4451130	0.6851399	0.5783919
	t=0.05	h^2_{pop}	0.2430760 (0.0497712)	0.2689810 (0.0487087)	0.4185690 (0.0659350)	0.6770240 (0.0813763)	0.5824480 (0.0785137)
		h^2_{kin}	0.0000009 (0.0384626)	0.0108812 (0.0331931)	0.0231210 (0.0545600)	0.0000009 (0.0718124)	0.0000009 (0.0625187)
		h^2_{pk}	0.2430769	0.2798622	0.4416900	0.6770249	0.5824489
	t=0.01	h^2_{pop}	0.2264140 (0.0374344)	0.2752630 (0.0404486)	0.4465800 (0.0469805)	0.6587400 (0.0542465)	0.5741510 (0.0560763)
		h^2_{kin}	0.0138312 (0.0182794)	0.0034573 (0.0135985)	0.0000010 (0.0226367)	0.0000009 (0.0296998)	0.0000009 (0.0240457)
		h^2_{pk}	0.2402452	0.2787203	0.4465810	0.6587409	0.5741519
	t=0.005	h^2_{pop}	0.2265440 (0.0357928)	0.2810670 (0.0396063)	0.4461100 (0.0441867)	0.6523690 (0.0507328)	0.5696410 (0.0533266)
		h^2_{kin}	0.0127770 (0.0139642)	0.0000009 (0.0091444)	0.0000010 (0.0161608)	0.0000010 (0.0188489)	0.0000009 (0.0155367)
		h^2_{pk}	0.2393210	0.2810679	0.4461110	0.6523700	0.5696419
	t=0.001	h^2_{pop}	0.2485630 (0.0353771)	0.2780550 (0.0385074)	0.4420260 (0.0420207)	0.6444640 (0.0484995)	0.5636970 (0.0514328)
		h^2_{kin}	0.0000009 (0.0080807)	0.0010970 (0.0049754)	0.0000010 (0.0085612)	0.0000010 (0.0051735)	0.0000009 (0.0036250)
		h^2_{pk}	0.2485639	0.2791520	0.4420270	0.6444650	0.5636979
Model 4		h^2_{ped}	0.2377390 (0.0382416)	0.3131680 (0.0431046)	0.4447200 (0.0472902)	0.6589970 (0.0582174)	0.5821470 (0.0606762)

Supplementary Table 3: h^2_{GRM} (h^2 with no thresholded GRM fitted), h^2_{pop} (the additive genetic variance explained by the full GRM (equivalent to h^2_{GRM} from model 1)), h^2_{kin} (the additive genetic variance explained by the thresholded GRM), h^2_{pk} (the sum of h^2_{pop} and h^2_{kin}) and h^2_{ped} (h^2 estimated using the pedigree) estimates for each polygenic trait for models 1, 3 and 4 with their respective standard errors.

		Neonate	Lamb				
		Birth weight	August weight	Foreleg length	Hindleg length	Metacarpal length	Jaw length
	t=0.05	0.09867	0.2286	0.08066	0.1525	0.2017	0.04067
Model 2	t=0.1	0.3461	0.4978	0.0813	0.1976	0.159	0.03831
	MAF=0.1	NA	NA	NA	NA	NA	NA
	MAF=0.05	NA	NA	NA	NA	0.4605	NA
	MAF=0.01	0.3194	0.1031	0.3295	NA	NA	NA
	MAF=0.005	0.1012	0.1945	0.2323	NA	NA	NA
Model 3	MAF=0.001	NA	NA	NA	NA	NA	NA

		Adult				
		August weight	Foreleg length	Hindleg length	Metacarpal length	Jaw length
	t=0.05	0.1643	0.06552	NA	NA	0.05255
Model 2	t=0.1	0.01435	0.003718	0.1067	0.3315	0.01074
	MAF=0.1	NA	NA	NA	NA	NA
	MAF=0.05	NA	0.3687	0.3361	NA	NA
	MAF=0.01	0.211	0.3964	NA	NA	NA
	MAF=0.005	0.1611	NA	NA	NA	NA
Model 3	MAF=0.001	NA	0.4046	NA	NA	NA

Supplementary Table 4: p values for the LRT for the inclusion of the thresholded GRMs for each threshold for models 2 and 3 for each polygenic trait. "NA" means that the model did not converge for that trait and threshold - we consider this to be equivelent to a p value of 1. Green highlights cells with a p value < 0.05

			Male horn type		Female horn type		Coat colour		Coat pattern	
Model 1		h^2_{GRM}	0.3518910	(0.0269923)	0.2282130	(0.0267518)	0.6132300	(0.0127196)	0.3499710	(0.0161968)
Model 2	t=0.05	h^2_{pop}	0.3177530	(0.0345399)	0.2174220	(0.0369595)	0.6054230	(0.0151641)	0.3587990	(0.0194481)
		h^2_{kin}	0.0583169	(0.0350713)	0.0151738	(0.0366140)	0.0130067	(0.0125519)	0.0000010	(0.0160213)
		h^2_{pk}	0.3760699		0.2325958		0.6184297		0.3588000	
	t=0.1	h^2_{pop}	0.3371430	(0.0319960)	0.2247330	(0.0338976)	0.6064820	(0.0146091)	0.3532080	(0.0182692)
		h^2_{kin}	0.0288222	(0.0315916)	0.0052941	(0.0323712)	0.0116538	(0.0113208)	0.0000010	(0.0140086)
		h^2_{pk}	0.3659652		0.2300271		0.6181358		0.3532090	
Model 4		h^2_{ped}	0.3905800	(0.0369544)	0.2387470	(0.0313989)	0.6529220	(0.0189878)	0.3333320	(0.0222770)

Supplementary Table 5: h^2_{GRM} (h^2 with no thresholded GRM fitted), h^2_{pop} (the additive genetic variance explained by the full GRM (equivalent to h^2_{GRM} from model 1)), h^2_{kin} (the additive genetic variance explained by the thresholded GRM), h^2_{pk} (the sum of h^2_{pop} and h^2_{kin}) and h^2_{ped} (h^2 estimated using the pedigree) estimates for each monogenic trait for models 1, 2 and 4 with their respective standard errors.

			Male horn type			Female horn type		
			Whole genome	Chromosome	1Mb either side of gene	Whole genome	Chromosome	1Mb either side of gene
Model 1		h^2_{GRM}	0.3518910 (0.0269923)	0.2505240 (0.0255399)	0.1294440 (0.0236076)	0.2282130 (0.0267518)	0.2555530 (0.0273251)	0.1993230 (0.0304379)
Model 3	t=0.1	h^2_{pop}	0.2919600 (0.0441054)	0.3011640 (0.0449449)	0.1231030 (0.0266039)	0.2498050 (0.0491979)	0.3736430 (0.0560876)	0.2051340 (0.0326240)
		h^2_{kin}	0.0648018 (0.0401937)	0.0000009 (0.0373498)	0.0219039 (0.0225205)	0.0000010 (0.0422002)	0.0000008 (0.0498471)	0.0000010 (0.0421791)
		h^2_{pk}	0.3567618	0.3011649	0.1450069	0.2498060	0.3736438	0.2051350
	t=0.05	h^2_{pop}	0.3526700 (0.0351791)	0.2915060 (0.0338265)	0.1305370 (0.0241229)	0.2308140 (0.0371107)	0.3068430 (0.0381716)	0.2025420 (0.0312843)
		h^2_{kin}	0.0000010 (0.0256721)	0.0000010 (0.0227651)	0.0000011 (0.0103728)	0.0000010 (0.0263063)	0.0000009 (0.0256807)	0.0000010 (0.0231875)
		h^2_{pk}	0.3526710	0.2915070	0.1305381	0.2308150	0.3068439	0.2025430
	t=0.01	h^2_{pop}	0.3501960 (0.0283281)	0.2538920 (0.0264127)		0.2308020 (0.0284570)	0.2607610 (0.0284598)	
		h^2_{kin}	0.0015435 (0.0111964)	0.0000010 (0.0054208)		0.0000010 (0.0107354)	0.0000010 (0.0057006)	
		h^2_{pk}	0.3517395	0.2538930		0.2308030	0.2607620	
	t=0.005	h^2_{pop}	0.3523240 (0.0276263)	0.2518410 (0.0259569)		0.2261640 (0.0275762)	0.2628470 (0.0282689)	
		h^2_{kin}	0.0000010 (0.0076378)	0.0000010 (0.0038393)		0.0025333 (0.0077549)	0.0000010 (0.0051967)	
		h^2_{pk}	0.3523250	0.2518420		0.2286973	0.2628480	
	t=0.001	h^2_{pop}	0.3520460 (0.0271412)	0.2535350 (0.0258705)		0.2281370 (0.0269024)	0.2613130 (0.0279011)	
		h^2_{kin}	0.0000010 (0.0032549)	0.0000010 (0.0033962)		0.0004658 (0.0027910)	0.0000010 (0.0049876)	
		h^2_{pk}	0.3520470	0.2535360		0.2286028	0.2613140	
Model 4		h^2_{ped}	0.3905800 (0.0369544)	0.3905800 (0.0369544)	0.3905800 (0.0369544)	0.2387470 (0.0313989)	0.2387470 (0.0313989)	0.2387470 (0.0313989)

			Coat colour			Coat pattern		
			Whole genome	Chromosome	1Mb either side of gene	Whole genome	Chromosome	1Mb either side of gene
Model 1		h^2_{GRM}	0.6132300 (0.0127196)	0.5229850 (0.0160838)	Failed to converge	0.3499710 (0.0161968)	0.2433330 (0.0192692)	Failed to converge
Model 3	t=0.1	h^2_{pop}	0.6666610 (0.0274822)	0.5728020 (0.0321004)		0.4370840 (0.0285590)	0.2884420 (0.0286982)	
		h^2_{kin}	0.0000009 (0.0281649)	0.0000011 (0.0350071)	Failed to converge	0.0000008 (0.0277818)	0.0000011 (0.0213478)	Failed to converge
		h^2_{pk}	0.6666619	0.5728031		0.4370848	0.2884431	
	t=0.05	h^2_{pop}	0.6406340 (0.0199443)	0.5640030 (0.0255429)		0.3860980 (0.0214723)	0.2544570 (0.0232076)	
		h^2_{kin}	0.0000010 (0.0177994)	0.0000011 (0.0246360)	Failed to converge	0.0000009 (0.0168216)	0.0000011 (0.0098138)	Failed to converge
		h^2_{pk}	0.6406350	0.5640041		0.3860989	0.2544581	
	t=0.01	h^2_{pop}	0.6232530 (0.0142494)	0.5370430 (0.0181533)		0.3544790 (0.0170833)	0.2595920 (0.0210876)	
		h^2_{kin}	0.0000011 (0.0071022)	0.0000012 (0.0114757)	Failed to converge	0.0000010 (0.0065411)	0.0000011 (0.0062941)	Failed to converge
		h^2_{pk}	0.6232541	0.5370442		0.3544800	0.2595931	
	t=0.005	h^2_{pop}	0.6207620 (0.0136306)	0.5291660 (0.0170116)		0.3508820 (0.0166385)	0.2514000 (0.0201258)	
		h^2_{kin}	0.0000011 (0.0052087)	0.0000012 (0.0073172)		0.0000010 (0.0043203)	0.0000011 (0.0048451)	
		h^2_{pk}	0.6207631	0.5291672		0.3508830	0.2514011	
	t=0.001	h^2_{pop}	0.6177950 (0.0129931)	0.5235020 (0.0161557)		0.3488810 (0.0162390)	0.2437710 (0.0193240)	
		h^2_{kin}	0.0000011 (0.0016679)	0.0000012 (0.0018069)		0.0000010 (0.0015172)	0.0000011 (0.0026712)	
		h^2_{pk}	0.6177961	0.5235032		0.3488820	0.2437721	
Model 4		h^2_{ped}	0.6529220 (0.0189878)	0.6529220 (0.0189878)	0.6529220 (0.0189878)	0.3333320 (0.0222770)	0.3333320 (0.0222770)	0.3333320 (0.0222770)

Supplementary Table 6: h^2_{GRM} (h^2 with no thresholded GRM fitted), h^2_{pop} (the additive genetic variance explained by the full GRM (equivalent to h^2_{GRM} from model 1)), h^2_{kin} (the additive genetic variance explained by the thresholded GRM), h^2_{pk} (the sum of h^2_{pop} and h^2_{kin}) and h^2_{ped} (h^2 estimated using the pedigree) estimates for each monogenic trait for models 1, 3 and 4 with their respective standard errors.

Grey indicates that the model was not ran due to an insufficient number of SNPs remaining after filtering to calculate the GRM from.

		Male horn type			Female horn type		
		Whole genome	Chromosome	1Mb either side of gene	Whole genome	Chromosome	1Mb either side of gene
	t=0.05	0.05253	Not run	Not run	0.3396	Not run	Not run
Model 2	t=0.1	0.1866	Not run	Not run	0.4342	Not run	Not run
	MAF=0.1	0.05662	NA	0.07261	NA	NA	NA
	MAF=0.05	NA	NA	NA	NA	NA	NA
	MAF=0.01	0.4473	NA	Not run	NA	NA	Not run
	MAF=0.005	NA	NA	Not run	0.3603	NA	Not run
Model 3	MAF=0.001	NA	NA	Not run	0.4267	NA	Not run

		Coat colour			Coat pattern		
		Whole genome	Chromosome	1Mb either side of gene	Whole genome	Chromosome	1Mb either side of gene
	t=0.05	0.1502	Not run	Not run	NA	NA	Not run
Model 2	t=0.1	0.1486	Not run	Not run	NA	NA	Not run
	MAF=0.1	NA	NA	NA	NA	NA	NA
	MAF=0.05	NA	NA	NA	NA	NA	NA
	MAF=0.01	NA	NA	NA	NA	NA	NA
	MAF=0.005	NA	NA	Not run	NA	NA	Not run
Model 3	MAF=0.001	NA	NA	Not run	NA	NA	Not run

Supplementary Table 7: p values for the LRT for the inclusion of the thresholded GRMs for each threshold for models 2 and 3 for each monogenic trait. "NA" means that the model did not converge for that trait and threshold - we consider this to be equivelent to a p value of 1. "Not run" means we did not run that model.
